# Performance of Problem-Based Learning Based Image Teaching in Clinical Emergency Teaching

**DOI:** 10.3389/fgene.2022.931640

**Published:** 2022-06-27

**Authors:** Xiaohong Xu, Yingcui Wang, Suhua Zhang, Fengting Liu

**Affiliations:** ^1^ Department of Rheumatology and Immunology, Qilu Hospital (Qingdao), Cheeloo College of Medicine, Shandong University, Qingdao, China; ^2^ Department of Education, Qilu Hospital (Qingdao), Cheeloo College of Medicine, Shandong University, Qingdao, China; ^3^ Department of Geriatrics, Qilu Hospital (Qingdao), Cheeloo College of Medicine, Shandong University, Qingdao, China; ^4^ Department of Emergency, Qilu Hospital (Qingdao), Cheeloo College of Medicine, Shandong University, Qingdao, China

**Keywords:** PBL teaching method, traditional teaching method, imaging diagnosis, clinical emergency, performance of PBL

## Abstract

At present, with the rapid increase of emergency knowledge and the improvement of people’s requirements for medical quality, the traditional teaching mode cannot fully meet the needs of emergency teaching in the new era. PBL is a project-based teaching that allows students to have a deeper understanding of content knowledge and to better apply what they have learned to their lives. This paper aims to improve the clinical emergency teaching mode by PBL teaching method, and improve the comprehensive ability of clinical emergency of medical students. This article proposes a problem-based PBL imaging teaching method, combining the characteristics and content of clinical emergency courses, focusing on students, highlighting the problem-solving process, and improving students’ creative thinking ability. To cultivate students’ interest in clinical learning, develop their self-learning ability, train their teamwork and communication skills, and cultivate their ability to set, question and solve questions, so as to promote medical students’ overall comprehensive ability to integrate specialized knowledge and clinical practice. In this paper, the PBL teaching method and the traditional teaching method of comparative experiments show that the PBL teaching method can more effectively highlight the characteristics of clinical emergency medicine teaching mode, and make full use of the limited emergency teaching resources, so as to improve the quality of clinical emergency teaching. Compared with the traditional teaching mode, the theoretical knowledge and clinical operation skills of medical students under the PBL teaching mode are improved by 13%, Autonomous learning ability, communication ability and creative thinking ability have also been relatively improved.

## 1 Introduction

With the rapid evolution of mankind from the large-scale industrial age to the information age and even the age of artificial intelligence, we are increasingly aware of the seriousness of the various challenges that physicians have to face. The unified teaching method based on classroom teaching is the most widely used teaching method in medical colleges and universities in China. The traditional teaching mode focuses on teaching knowledge, although it can cultivate medical students with solid basic theoretical knowledge, but the lack of clinical practice ability and problem-solving ability will cause the phenomenon that theoretical knowledge and practical operation ability are divorced, so it is difficult to adapt to the clinical practice process. A relatively single teaching method often loses students’ interest and independent thinking ability, making teachers’ guidance and students’ learning burdens heavier and more difficult. Therefore, in clinical emergency education, in addition to transferring knowledge, it is more important for students to learn independent thinking, literature review, and the ability to realize autonomous learning, and to improve students’ ability to explore and solve problems, so as to cultivate high-quality suitable for the development of the times Medical talents. At the same time, this teaching adapts to the trend of modern teaching and the development of emergency medicine. Applying this model not only enables medical students to quickly grasp the relevant knowledge points, but also cultivates their learning ability and improves their ability to effectively apply their knowledge when dealing with clinical problems.

Although originally designed as a teaching method for graduate admissions in medicine, problem-based learning (PBL) has been widely used in undergraduate medicine, science, and social science courses. Although it is generally acknowledged that new learners of PBL need support, this does not provide a good description of the undergraduate course, which makes some students feel incomprehensible. In this submission, Moro C provides many extensive considerations and practical suggestions to support the transition of learners to PBL and universities. In a globalized world where the diversity of learners is increasing, this kind of support is particularly important in a learner-centered and socially responsible higher education pattern. But his research is not comprehensive enough, and more in-depth research is needed to prove the feasibility of this method (Moro and Mclean, 2017). The formative assessment of clinical teaching for emergency medicine (EM) teachers is limited. The purpose of this study is to develop a behavior-based tool to assess EM teachers’ clinical teaching skills during shift and provide feedback. Dehon e uses a three-stage structured development process. In the first stage, the nominal group technology is used to communicate with a group of teaching staff, and then with residents to generate potential assessment projects. Phase 2 includes an independent focus group and uses improved Delphi technology to evaluate the projects generated in phase 1 with faculty and residents as well as a group of experts. Since then, residents have divided the programs into novice, intermediate and advanced educator skills. Once the items to be included are identified and then ranked, the investigators build them into the tool (phase 3). Results: the final tool “teacher shift card” is a behavior anchored assessment and feedback tool to facilitate feedback to EM teachers on their teaching skills during the shift. However, his method is not supported by specific experimental data and lacks accuracy of experimental results (Dehon et al., 2018). In the past 20 years, social work education has built a bridge between the classroom and the scene by learning clinical skills through customer simulation. Pecukonis EV outlines an innovative simulation model, combined with LS, for teaching motivation interview (MI). In addition, he also discussed the guiding principles and specific steps of using simulation and LS to teach MI. Unfortunately, most simulation models ignore the method of real-time guidance and monitoring students. In the implementation of clinical simulation, there are few opportunities to correct students’ behaviors or practice new skills at the best teachable time in the interview. This instruction must wait until the end of the interview and the beginning of the presentation. With the addition of LS, the students’ simulation experience has been enhanced, because the tutor is now an active participant in the interview. With LS, teachers can now direct and even simulate appropriate clinical responses and interventions. However, the research model is too complex to be put into practical application (Yan, 2017; Pecukonis, 2021; Ramadan, 2021).

The innovation of this article lies in the advantages of the PBL teaching method, breaking through the traditional learning method, giving full play to the student-centered teaching method, cultivating students’ interest and ability in autonomous learning, and improving the ability of autonomous learning. The article also integrates integrated cognitive activities such as questioning, judging, comparing, choosing and analyzing, synthesizing and generalizing knowledge. ability. Ability to think, solve problems, and solve problems; In order to improve the quality of clinical emergency teaching, it is necessary to cultivate students’ teamwork ability, expand their knowledge, and master professional clinical emergency operation skills.

## 2 Implementation of Problem-Based Learning Based Image Teaching Method in Clinical Emergency

### 2.1 Problem-Based Learning Teaching Method

PBL is a problem based learning (PBL) which was founded by Howard Barrows, an educator in 1969 at the Medical College of McMaster University in Canada. That is, problem-based learning is a student-centered, group oriented and teacher oriented learning method (Umami, 2018; Liu, 20192020). The PBL model originated in medical education and is an inquiry-based teaching method, that is, a core learning approach for research-based learning, integrated practice courses. This method is in the form of group discussion in a complex and substantive problem situation, so that students can solve problems through independent research and cooperation, and learn and obtain the scientific knowledge behind the problems, so as to make it possible. It is a kind of education method combining basic science and clinical practice. The biggest feature of this teaching method is to change the passive learning of traditional teaching methods into active learning (Petruk, 2017; Gallagher et al., 2017). According to current research, in this teaching method, students are first grouped, and then questions are set. Under the guidance of teachers, each group of students discusses and analyzes to find answers to the problems. In the teaching process, firstly, students learn the professional theoretical knowledge more deeply by searching for answers on their own; secondly, through multiple rounds of group discussion and summarization, they have exercised their learning ability, communication skills and teamwork ability, and finally students deal with the actual situation. The ability to question can also be greatly improved (Stephanie and Hughes, 2016; Tarling et al., 2016). The traditional teaching method (Lecture-Based Learning), that is, the method of “learning based on teaching,” referred to as LBL (Light and Razak, 2020; Xu et al., 2022), is a traditional theoretical teaching method based on the teacher’s teaching (Ananga, 2020). It is an education-based teacher-centered teaching method that emphasizes the transfer of knowledge in the classroom. Cannot meet the needs of students for knowledge and practice, development skills are not enough to improve students’ ability to explore and solve problems (Dzombak et al., 2020; Posteraro et al., 2020). PBL education is a new educational strategy that allows students to find effective solutions to situational problems. This educational strategy is very consistent with the current theoretical theories and educational principles (Macqueen et al., 2016). In the medical field, the PBL medical teaching model is a process in which medical students work in small groups, with the participation and guidance of a tutor, to formulate, discuss and learn about a complex, multi-scenario, real-world problem-based topic or case.

### 2.2 Interpretation of Low-Quality Medical Images With Deep Learning Methods

#### 2.2.1 Image Degradation Mechanism

Assuming that A is a low-quality image and B is a corresponding high-quality image, the image degradation mechanism can be expressed as follows:

A=σ(B). (1)

Among them, σ represents the image degradation function in a broad sense, such as additive noise, multiplicative noise, compound noise, etc.

#### 2.2.2 Deep Learning Image Restoration Paradigm

Low quality images arise because of the presence of many unnecessary or redundant interfering information in the image data, which must be corrected prior to image enhancement processing and classification processing. The image restoration model based on deep learning can be expressed as a regression paradigm of supervised learning: assuming that the network model is M, it represents the modeling function of the image degradation mechanism, which can be regarded as the optimal approximation to the inverse function $\sigma^{-1}$ ; The input of is the low-quality image A, and the output result is expressed as M(A). The restoration requires that M(A) be as close as possible to the corresponding high-quality image Y. Training the recovery network can be seen as optimizing the following loss function:

$$\frac{\arg\min}{M}+\frac{1}{2}M\left(A\right)-B^{2}.$$ (2)

The above loss function generally only considers the mean square error of the gray values of the pixels corresponding to the restored image M(A) and the high-quality image B. Representative algorithms include MLP, CNN, etc. In addition to directly learning high-quality images, some also link residual learning and restoration, directly learning the noise distribution of the image, and the regression paradigm can be expressed as the following form:

$$\frac{\arg\min}{M}+\frac{1}{2}M\left(A\right)-\sigma^{2}.$$ (3)

Among them, M(A) is the image noise fitted by the network, which is the real image noise, which can be obtained by the following calculation

φ=A−B, (4)

That is, the difference between a low-quality image and a high-quality image. Compared with directly learning high-quality images, learning the noise distribution makes the optimization of the network easier, but to obtain the final restored image N, a further subtraction operation is required:

N=A−M(A). (5)

We can basically get the recovered image by the above subtraction operation, but we need to enhance the image in order to fully obtain the information in the image.

### 2.3 Gradient Regularization Image Enhancement Model

The results of image processing depend heavily on the mathematical model developed. Even for the same image, the results obtained using different mathematical models may vary greatly. Therefore, in the early stage of image restoration, people generally use known *a priori* information to build a gradient model.

#### 2.3.1 Gradient Regularization Image Restoration Paradigm

$$\frac{\arg\min}{M}+\frac{1}{2}M\left(A\right)-B^{2}+\frac{\rho }{2}t⊗M\left(A\right)-B^{2},$$ (6)

Where t is the gradient operator, which represents the convolution operation, and is the balance factor. It can also be simplified to:

$$\frac{\text{argmin}}{M}+\frac{1}{2}M\left(A\right)-B^{2}+\frac{\rho }{2}t⊗{\left(M\left(X\right)-B\right)}^{2}.$$ (7)

### 2.4 Heuristic Information

Image enhancement is a widely used digital image processing technique, and currently, there are many methods used for image enhancement. As with other image restoration methods, since there is a corresponding image as a reference, the method mentioned above is no longer used here, and the calculation formula is as follows:

$$10{\text{ln}}_{10}\left(\frac{MAX^{2}}{MSE\left(C,Z\right)}\right).$$ (8)

MAX represents the maximum gray value of the image. The image similarity is measured from three aspects: brightness, contrast, and structure. The calculation formula is as follows:

$$k\left(v,e\right)=\frac{2\eta_{v}\eta_{e}+x_{1}}{\eta_{v}^{2}+\eta_{e}^{2}+x_{1}}.$$ (9)

$$j\left(v,e\right)=\frac{2\sigma_{v}\sigma_{e}+x_{2}}{\sigma_{v}^{2}+\sigma_{e}^{2}+x_{2}}.$$ (10)

$$i\left(v,e\right)=\frac{\sigma_{ve}+x_{3}}{\sigma_{v}\sigma_{e}+x_{3}}.$$ (11)

SSIM(v,e)=k(v,e)+j(v,e)+i(v,e). (12)

Among them, $\eta_{v}$ , $\eta_{e}$ represent the mean value of the restored image v and the reference image e, $\sigma_{v}$ , $\sigma_{e}$ represent the standard deviation of v, e, $\sigma_{ve}$ represents the covariance between the two, $x_{1}$ , $x_{2}$ , $x_{3}$ are smoothing constants, used To avoid the situation where the denominator appears to be 0, the value is generally entered as follows:

$$x_{1}={\left(H_{1}\times G\right)}^{2}.$$ (13)

$$x_{2}={\left(H_{2}\times G\right)}^{2}.$$ (14)

$$x_{3}{=}_{2}^{x_{2}}.$$ (15)

Generally, $H_{1}=0.01,H_{2}=0.03$ , and L represent the maximum value of the image gray scale, and the value range is [0,1,0,]. The smaller the value, the lower the image distortion. The higher the image distortion rate, the higher the risk of introducing information or components that do not exist in the original image, which can have serious consequences, such as the addition of tissue components to biomedical images, which can affect the doctor’s diagnosis of the condition.

## 3 Experiments Based on Problem-Based Learning Teaching Method in Clinical Emergency

### 3.1 Experiment Object

Using the cluster sampling method, 50 students who practiced in a tertiary A hospital were used as the control group and received traditional teaching methods; 48 students who practiced in the emergency department were used as the experimental group and received PBL imaging teaching methods. After the teaching, the theory and skills of the two groups of students were evaluated; before and after the teaching, the two groups of students used self-study assessment tools, Skala Attitudes Skills Communication Skills and the Chinese version of the Critical Thinking Scale (Gruppen, 2017; Messman et al., 2020). According to the internship arrangement, evaluate and statistically analyze the evaluation results, and include all interns in the internship evaluation form. And before entering the emergency internship, at the end of the departmental internship, the comprehensive examination transcript will be evaluated, and the evaluation results will be statistically and data analyzed. The general information of the experimental group and the control group was compared with the pre-hospital assessment scores, and the difference was not statistically significant (*p* > 0.05), which was comparable.

The control group used traditional teaching methods, centered on the instructor, and intensively taught once a week. The content of the course included two basic elements: theoretical knowledge and technical skills. One week before the end of the internship, the trainees will be evaluated based on theory and skills.

The experimental group adopts the PBL teaching method, emphasizing the student-centered approach, integrating clinical case group discussions and simulated clinical scenarios into the teaching process, allowing students to analyze cases and find answers, thereby improving their own abilities. In this kind of curriculum construction, we break the content arrangement of the disease as the outline, teaching content as the outline of symptoms, and set the common clinical symptoms of emergency medicine, such as: dyspnea, chest pain, abdominal pain, coma, etc.

### 3.2 Teaching Preparation

Improve clinical education resources: establish and improve the clinical literature search network in hospitals, emergency departments, and student cards (Kim et al., 2018; Brown, 2021). Strictly select teachers with lessons: select clinical education teachers and create a clinical education team in the emergency department. The team is evaluated and approved by the education and research department of the emergency department, and selects emergency educators with solid basic theoretical knowledge, professional knowledge and awareness, responsibility and communication skills, and excellent knowledge dissemination skills (Dull et al., 2018).

### 3.3 Draw Up an Education Plan

Complete the training content of the teachers in the experimental group, and determine a unified teaching plan based on the mastery of the relevant PBL teaching concepts and methods, and the syllabus, purpose, requirements, and content of the clinical emergency teaching course. Using multimedia to explain the concept of PBL teaching method and the teaching method based on question and discussion (Dehon et al., 2019; Karki, 2020; Tabassum et al., 2022), organize and teach teachers to discuss methods of mobilizing students’ enthusiasm. Determine the PBL teaching content, such as case selection for frequently-occurring diseases that are more practical. Complete the PBL problem design PBL teaching cases are developed with the most typical emergency and frequently-occurring disease as the core, establish poorly structured problems, and complete the PBL problem design. Prepare 3 cases, each batch of students in the emergency department are required to complete one case, each case is divided into three times, each case has three acts, each act is arranged for independent study (2 days), concentrated discussion (1 class hour), and Group summary (1 class hour).

### 3.4 Draw Up a Training Plan

Pre-admission training control group for students: explain the syllabus, purpose, content, and assessment requirements of clinical emergency medicine students, and distribute critical thinking survey forms and questionnaires on clinical medicine students’ cognition and attitude towards PBL. Emphasize that during the clinical emergency internship, strictly abide by the rules and regulations of clinical emergency (Levine et al., 2016; Bertino et al., 2021). Experimental group: Explain the syllabus, purpose, content and assessment requirements of clinical emergency medicine students, and distribute critical thinking survey forms and questionnaires on medical students’ cognition and attitude towards PBL. Then carry out the training before the implementation of PBL, that is, explain the concept of the PBL teaching model, the implementation method, the purpose of implementing PBL in the clinical emergency department, and the main points of cooperation. Distribute PBL teaching cases to allow students to preview in advance and ask students to analyze and discuss the cases, prepare and record their presentations.

Pre-admission training control group for students: based on traditional knowledge transmission, the one-to-one teaching method, namely LBL teaching method, is based on the traditional knowledge transmission, the teaching teacher implements the teaching of clinical emergency students according to the internship syllabus and requirements. Teaching tasks, first review relevant theoretical knowledge, combined with common clinical emergency diseases, students complete the internship tasks in the emergency department under the leadership of the teacher. Experimental group: PBL + LBL teaching method is adopted. The specific implementation methods are as follows: 1) Self-directed learning: After entering the course, students can find the best arguments and answers through reading textbooks, searching literature, consulting teachers, etc. in combination with relevant questions raised in the case, and can share the collected evidence with each other. One student recorded his doubts or the answers he found, and reported when preparing for group discussion (Ahn et al., 2016; Runde et al., 2016). Also ask students to think about: ① How to consider the changes in the patient’s condition? ②Try to analyze the causes and mechanisms of the above changes in the patient’s condition? ③The current diagnosis? ④Current management? 2) Organizing discussion: Arrange students to report the results of the group study, ask questions based on actual clinical cases, the students report and speak, use the brainstorming method, each student puts forward their own opinions, and teaches the teacher to supplement the scenes involved in the problem and further ask questions, The team leader will make a record, and the team leader will list the best answers to the questions after the discussion (Choi et al., 2019; Jain et al., 2020). In the course of the experiment, the teacher is responsible for coordinating, motivating them to actively participate in the discussion, ensuring that the discussion direction is targeted at the teaching purpose, allowing students to understand the clinical manifestations of patients during the discussion process, learn the key points of knowledge such as communication skills with patients, strengthen students’ understanding and mastery of clinical emergency operation in the emergency department, and improve clinical thinking and problem-solving ability (Sengan, Khalaf, Rao, Sharma, Amarendra, Hamad; Jain et al., 2021). 3) Summarize: After the discussion, the teacher and students will jointly summarize the characteristics of clinical emergency department, the concept of common diseases, clinical diagnosis and precautions. At the same time, students are required to complete a reflection diary and sort out the ideas for solving problems to promote Theory and practice (Patidar and Pichholiya, 2016; Tabassum et al., 2017).

### 3.5 Data Processing and Statistical Analysis Methods

Use EXCEL software to make a unified data entry template. After a researcher has verified that the questionnaire is correct twice, the content of the questionnaire is recorded in the data template, and the survey data is statistically analyzed with SPSS 20.0 (Hexom et al., 2017), test level *α* Set to 0.05, the *p* values of the two probabilities are statistically significant, so *p* < 0.05. 1) The measured data is expressed by (±S), and the measured data is expressed by frequency or percentage. 2) For the measurement data that satisfies the smoothness and uniformity of the variance, please use two independent t-test samples, and use the chi-square test to measure the data between the two groups. For measurement data that does not satisfy the smoothness and uniformity of the variance, the rank sum test is used for the total score data, and the comparison between two or more groups is performed using ANOVA. 3) Use factor analysis and stepwise regression analysis to analyze the factors that affect critical thinking ability. 4) When comparing the emergency clinical ability before and after baseline, 2 weeks later, and 1 month later, the paired sample test is used for volume data, and the second test is used for measuring data. *p* < 0.05 is considered to be statistically significant.

### 3.6 Problem-Based Learning Teaching Implementation Plan Process in Clinical Emergency

**FIGURE 1:**
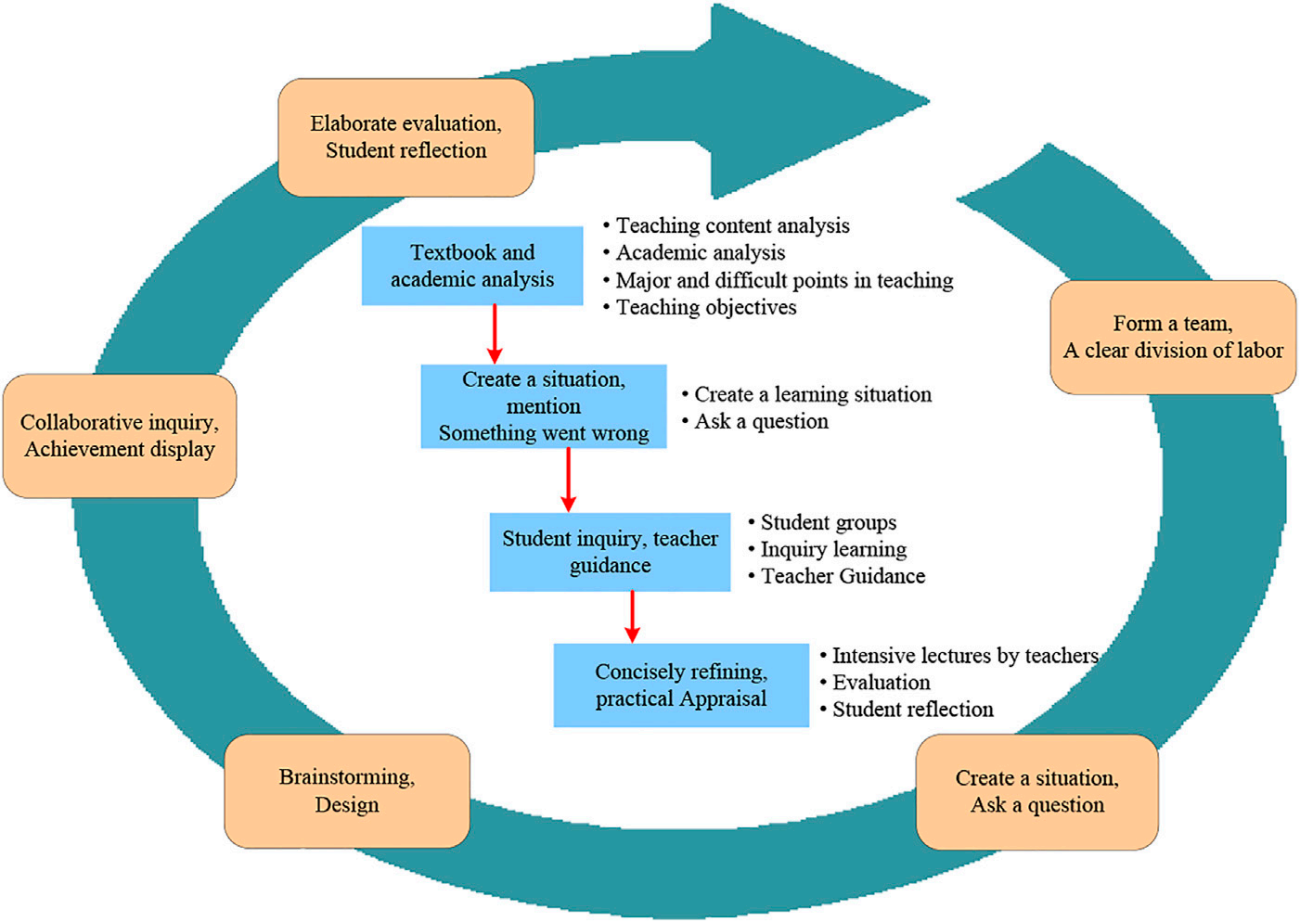
Emergency department PBL teaching implementation plan process.

The implementation plan based on the PBL teaching method in clinical emergency teaching is shown in Figure 1. The clinical emergency PBL teaching method experiment is completed according to the process, and then compared with the traditional teaching method, the experimental results are obtained.

**FIGURE 2:**
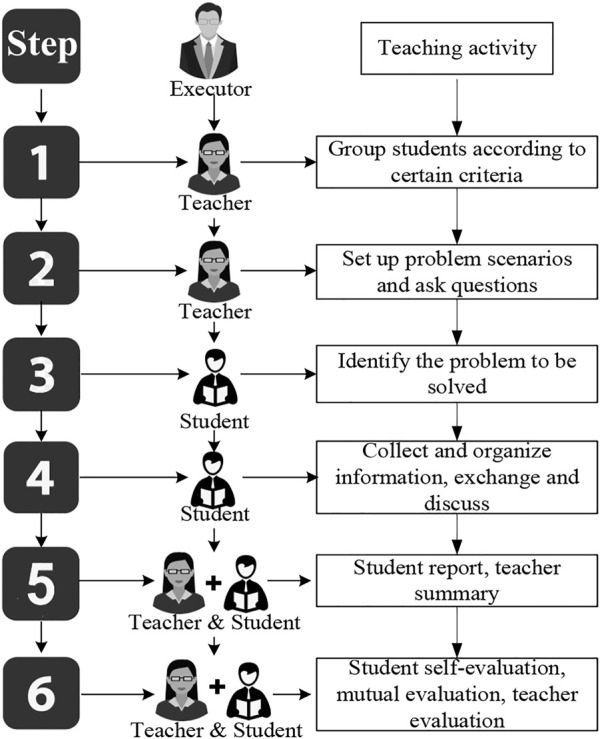
The teaching process of PBL teaching mode.

The PBL teaching mode is a problem-based teaching mode with students as the core. The teaching process is shown in Figure 2:

## 4 Performance Analysis Based on Problem-Based Learning Imaging Teaching Method in Clinical Emergency Teaching

### 4.1 Basic Information of the Two Groups of Students

As shown in Table 1, the experimental group and the control group were similar in age, basic average academic performance of professional courses, and the composition ratio of men and women, and the difference was not statistically significant (*p* > 0.05).

**TABLE 1:** Comparison of two basic data.

Project	Test group	Comparison group	t	*p*
Age	22.07 ± 0.47	21.75 ± 0.59	0.584	0.621
Grades	82.15 ± 3.75	82.47 ± 4.03	0.056	0.852
Gender composition (M/F)	15/33	18/32	0.042	0.781

### 4.2 Comparison of Problem-Based Learning Experimental Courses and Overall Attitudes

The basic abilities of students’ PBL are scored by five levels of 1–5. As shown in Table 2, there is no statistical difference between the scores of the two groups of students (*p* > 0.05).

**TABLE 2:** Comparison of cognition and attitude of internship in PBL.

Project	Test group	Comparison group	t	*p*
PBL understanding	4.13 ± 0.51	3.88 ± 0.46	−1.036	>0.05
PBL participation	3.76 ± 0.19	3.45 ± 0.21	−0.974	>0.05
Willingness to participate in PBL	3.84 ± 0.42	2.79 ± 0.28	−1.482	>0.05
The need to participate in PBL	4.25 ± 0.37	3.15 ± 0.52	−1.306	>0.05

As shown in Table 3, the experimental group and the control group have statistically significant differences in the experimental classes, satisfaction, sense of responsibility, and confidence in future work (*p* < 0.05). It can be seen that the scores of the students in the experimental group are higher than those in the control group. Therefore, it can be concluded that the overall attitude of the students in the experimental group is better than that of the control group.

**TABLE 3:** Comparison of the overall attitudes of the two groups of students to the experimental class.

Project	Test group	Comparison group	t	*p*
Whether the experimental class is important or not	2.56 ± 0.72	2.18 ± 0.45	8.163	<0.05
Satisfaction with the profession	2.64 ± 0.61	2.06 ± 0.53	4.581	<0.05
The sense of responsibility to study hard	2.58 ± 0.64	2.22 ± 0.41	9.742	<0.05
Confidence in future clinical work	2.77 ± 0.78	2.17 ± 0.38	7.985	<0.05

### 4.3 Teaching Situation

Two independent sample tests were used to compare the clinical ability scores of the two groups of students during two and 4 weeks of teaching. The result is shown in the figure below:

As shown in Table 4, except for the dimension of clinical handling ability (*p* > 0.05), the scores of the PBL teaching experiment group and the total score of emergency clinical ability were higher than those of the control group, the difference was statistically significant (*p* < 0.05) As shown in Table 5, after 4 weeks of teaching, except for the clinical management ability, the scores and total scores of the clinical emergency ability of the PBL teaching group were significantly higher than those of the control group, and the difference was statistically significant (*p* < 0.05).

**TABLE 4:** Results of emergency clinical ability of the two groups after 2 weeks of teaching.

Evaluation index	Test group	Comparison group	t	*p*
Clinical treatment	6.03 ± 0.41	5.92 ± 0.37	0.71	>0.05
Communication and coordination	5.12 ± 0.39	4.95 ± 0.57	2.34	<0.05
health education	6.41 ± 0.52	5.76 ± 0.39	4.85	<0.05
Nursing Research	8.14 ± 0.47	7.27 ± 0.46	1.55	<0.05
Clinical teaching	7.35 ± 0.39	6.82 ± 0.27	3.89	<0.05
Clinical management	6.72 ± 0.48	6.34 ± 0.19	7.63	<0.05
Mental quality	6.85 ± 0.51	5.73 ± 0.41	5.43	<0.05

**TABLE 5:** Results of emergency clinical ability of the two groups after 4 weeks of teaching.

Evaluation index	Test group	Comparison group	t	*p*
Clinical treatment	7.21 ± 0.39	6.58 ± 0.42	0.76	<0.05
Communication and coordination	6.04 ± 0.43	5.62 ± 0.37	3.15	<0.05
health education	6.93 ± 0.52	6.12 ± 0.39	5.21	<0.05
Nursing Research	9.25 ± 0.47	7.94 ± 0.46	2.33	<0.05
Clinical teaching	7.89 ± 0.39	7.29 ± 0.27	4.15	<0.05
Clinical management	7.06 ± 0.48	7.21 ± 0.19	6.53	<0.05
Mental quality	7.62 ± 0.51	6.31 ± 0.41	4.86	<0.05

**FIGURE 3:**
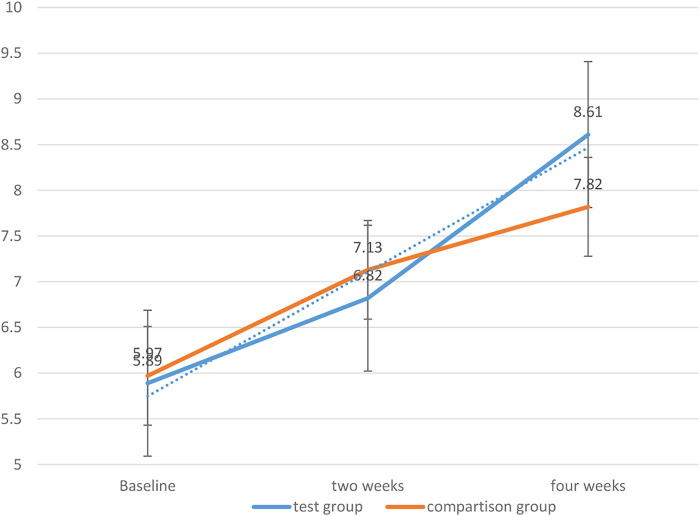
Trends in emergency clinical capabilities.

Figure 3 depicts the trends of the clinical emergency ability of the two groups of students with the internship time. The results show that the two groups of students have improved with the elapse of internship in the emergency department, but the PBL group has a larger increase than the control group. It is statistically significant (*p* < 0.05). It can be seen that the PBL teaching method has a greater improvement in clinical emergency ability.

In this experiment, repeated measures analysis of variance was used to compare the clinical first aid capabilities of the two groups of trainees after 2 and 4 weeks. The between-group effects are divided into different groups (i.e., PBL teaching group and control group), the interaction effect is the grouping time, and the between-group effects are (2 weeks, 4 weeks).

The results are shown in Table 6: 1) Except for the clinical management dimension, *p* is less than 0.05 for the inter-group effects, indicating that the differences in the overall mean values of the various dimensions of clinical emergency ability in different groups are statistically significant, that is, PBL teaching can improve students. 2) The intra-group effect *p* < 0.05 means that the difference in the overall mean scores of the various dimensions of the clinical emergency ability and the overall average of the total score is statistically significant. With the change of time, the clinical emergency ability of the trainees has improved. 3) Except for the clinical management ability, the interaction effect is less than 0.05, which means that the interaction effect of different measurement time and group has statistical significance on the overall mean difference of the various dimensions of emergency clinical ability and the total score, indicating that 4 weeks of PBL teaching can significantly improve the clinical performance of students Emergency room capacity.

**TABLE 6:** Repeated measures analysis of variance of two groups of clinical emergency ability score.

Project	Between-group effects		Within-group effect		Interaction effect	
	F	*p*	F	*p*	F	*p*
Clinical treatment	18.64	<0.05	121.34	<0.05	8.53	<0.04
Communication and coordination	82.48	<0.05	184.14	<0.05	19.14	<0.05
health education	23.76	<0.05	97.39	<0.05	34.76	<0.05
Nursing Research	80.47	<0.05	68.25	<0.05	18.15	<0.05
Clinical teaching	43.85	<0.05	74.47	<0.05	21.84	<0.05
Clinical management	41.86	<0.05	66.19	<0.05	50.59	<0.05
Mental quality	78.81	<0.05	71.84	<0.05	19.18	<0.05
Total score	369.87	<0.05	683.62	<0.05	172.19	<0.05

### 4.4 Comparison of Ratings of Different Teaching Methods Between Two Groups of Students

**FIGURE 4:**
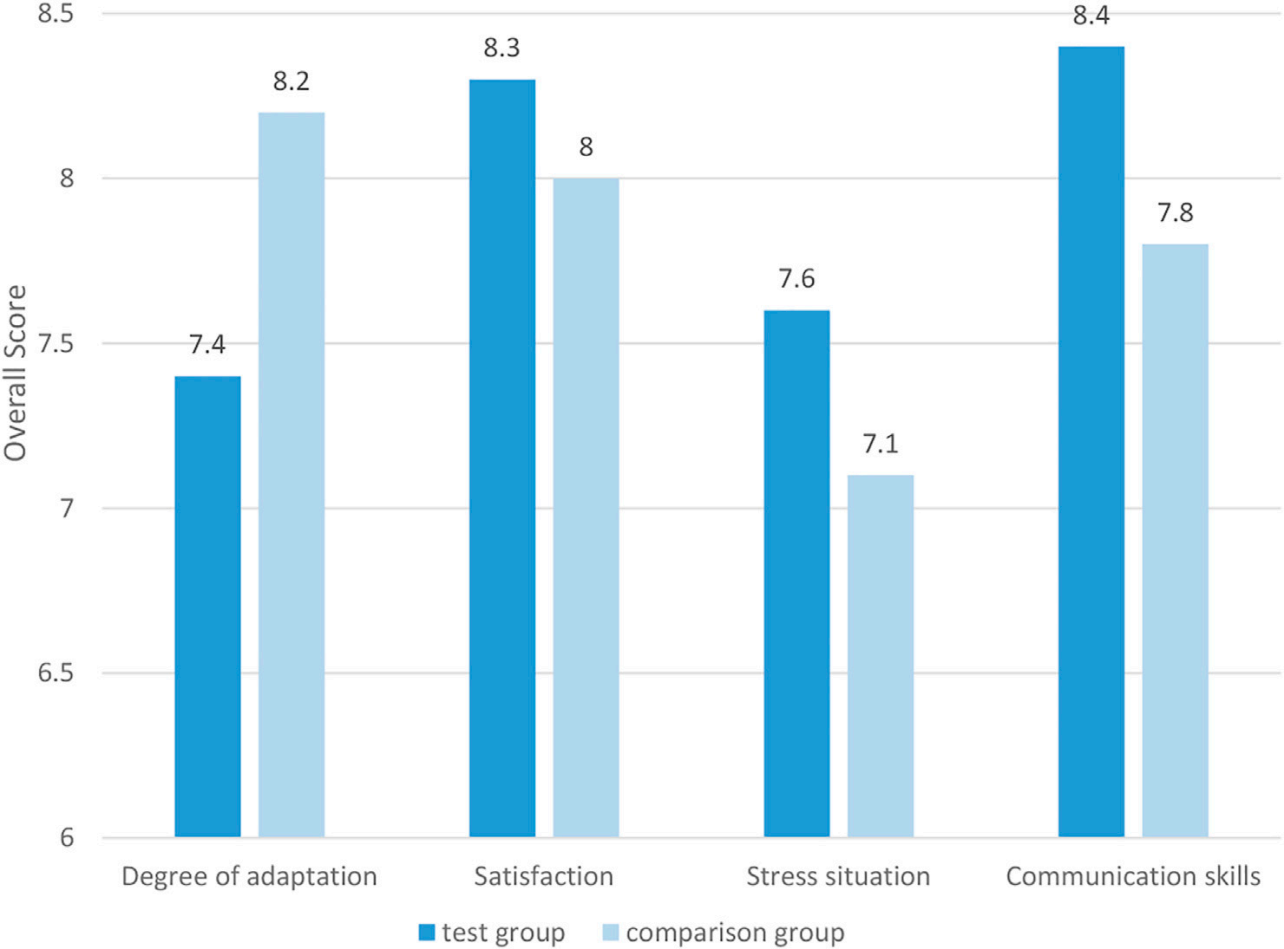
Scores for all aspects of two different teaching methods.

The scoring system uses a 10-point system to score the adaptability, satisfaction, pressure, and improving communication skills of the teaching method. The results are shown in Figure 4.

As shown in Figure 4, there are statistically significant differences between the two groups of students in terms of their degree of adaptation, satisfaction, stress, and improving their communication skills (*p* < 0.05). From the perspective of adaptation, The overall adaptation rate of the experimental group is lower than that of the control group. Therefore, the students in the experimental group are less adaptable to the PBL teaching method than the control group students’ adaptation to the traditional teaching method. From the perspective of stress, the experimental group’s pressure score is higher. Therefore, the PBL teaching method brings more pressure to the study of the experimental group students than the traditional teaching method to the control group of students. From the perspective of satisfaction and the improvement of personal communication skills, the scores of the experimental group are greater than those of the comparison group, so the PBL teaching method improves personal communication skills more than the traditional teaching method.

**FIGURE 5:**
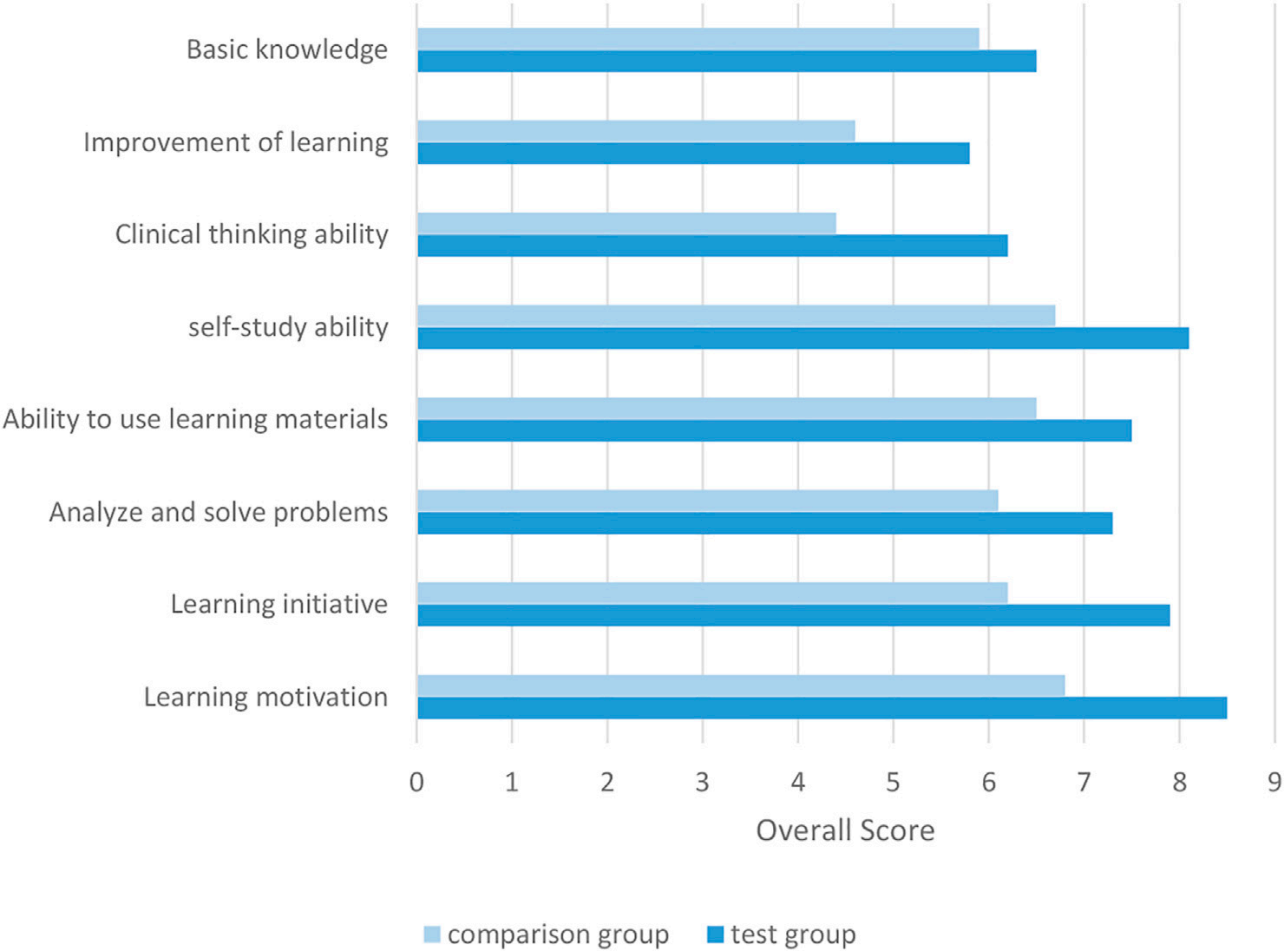
Comparison of the improvement of the two groups of students’ learning ability by two different teaching methods.

As can be seen in Figure 5, the results show that the two different teaching methods give two groups of students a statistically significant difference in the level of cultivation of various abilities, *p* < 0.05, and the improvement rate of the experimental group is greater than that of the control group. Therefore, the PBL teaching method gives The increase in learning ability brought by the experimental group students was more significant than the increase in learning ability brought by the traditional teaching method to the control group.

### 4.5 Comparison of the Two Groups of Students’ Theoretical and Operational Performance

The independent sample test was used to analyze the theoretical scores of the two groups of students. The results are shown in Table 7. The test scores of the students in the experimental group were higher than those in the control group, and the average scores in the clinical experiments were also higher than those of the control group. The difference was statistically significant (*p* < 0.05). It can be seen that The PBL teaching method has a greater improvement in theoretical and operational performance.

**TABLE 7:** Comparison of experimental results.

Project	Test group	Comparison group	t	*p*
Theory test	88.67 ± 1.786	82.43 ± 1.658	7.541	<0.05
Operational exam	87.46 ± 1.457	85.79 ± 1.514	8.647	<0.05

### 4.6 Comparison of the Questionnaire Received by the Two Groups of Students After Teaching

Table 8 shows the comparison of the effects of the experimental group and the control group after teaching. The results of the questionnaire show that the special gains of the experimental group after teaching are greater than those of the control group, except for the improvement of innovation ability, which is not statistically significant (*p* > 0.05). The improvement of self-learning ability was the biggest difference between the experimental group and the control group, and the differences in other aspects were statistically significant (*p* < 0.05).

**TABLE 8:** Comparison of the two groups of students after teaching.

Project	Type	Test group	Comparison group	t	*p*
Improved language organization	Y	31	18	8.457	<0.05
	N	17	32		
Improved logical thinking ability	Y	29	14	10.716	<0.05
	N	19	36		
Improved innovation ability	Y	16	15	0.974	>0.05
	N	32	35		
Improve self-learning ability	Y	40	10	12.385	<0.05
	N	8	40		

## 5 Conclusion

The PBL teaching method can fully exercise people’s thinking skills such as decision-making, creativity, reasoning and analysis in the process of conceiving solutions, exploring independently, making decisions and seeking solutions to problems. This article is based on the PBL teaching method to construct a clinical emergency teaching plan and carry out experimental application. In contrast to traditional teaching methods, the clinical emergency PBL teaching method constructed in this article is more prominent in improving the performance of students’ clinical emergency ability, and can more effectively improve students’ theoretical technical operation level, autonomous learning ability, communication ability and communication judgment ability. Thinking ability has obvious advantages in experimental teaching. It is more conducive to cultivating medical students’ good study habits, enthusiasm and the ability to make full use of existing learning resources, thereby improving the comprehensive clinical professional quality of medical students. In the operation skills learning in the medical emergency, the PBL teaching method makes the learning goals and meanings of medical students clearer. The important role of various skills operations has been verified in the implementation process, and the proactiveness of the operation skills practice has been improved. However, there are still some shortcomings in this research. The traditional teaching model is deeply ingrained. A small number of teachers and students have a certain degree of rejection to the difficulty of adapting to the PBL teaching method. There is no multi-factor analysis on the factors that affect the effect of experimental teaching. Further research is needed. In the future article, we will focus on the factors that affect the effectiveness of PBL teaching practice from several levels.

## Data Availability

The original contributions presented in the study are included in the article/supplementary material, further inquiries can be directed to the corresponding authors.
